# Report of Five Laparotomies

**Published:** 1890-10

**Authors:** X. O. Werder

**Affiliations:** Pittsburg, Pa.


### REPORT OF FIVE LAPAROTOMIES.

By X. O. WERDER, M. D., PITTSBURG, PA.

CASE I.—Intraligamentous cyst. Aged 22 years; epileptic; typhoid fever in October last, followed by bad health. Noticed a tumor growing since, reaching from one to one and one-half inches above the umbilicus down into the vagina, bulging out Douglas' *cul de sac* and pushing down the anterior wall of the rectum, in fact almost completely filling out the pelvic cavity. The tumor was immovable, fluctuating and distinctly pulsating both over the abdomen and in the vagina, suggesting the possibility of an aneurism. Exploratory laparotomy, March 29th, exposed an intraligamentous cyst, which had to be peeled out of its capsule. It was a tedious and difficult task. Drainage. Patient made an excellent recovery, without rise of temperature and notable increase of pulse rate. Had an epileptic attack immediately after operation, then none for a week, though previous to operation she had a number of them every day. After first week they returned at intervals of several days.

CASE II.—Pyosalpynx. Miss M. D., 26 years of age, had poor health for several years; about a year ago she was obliged to go to bed, when an abscess ruptured into her rectum, which continued to discharge for several months. After the discharge had ceased, her health improved and she gained flesh, but six weeks later she experienced great pain in her right side, and when she entered the Mercy hospital another abscess had ruptured into the vagina. She was extremely emaciated and anæmic. Laparotomy performed April 6th. The anæsthetic used was the "mixture." Immediately after opening the peritoneal cavity she became asphyxiated. Respiration and pulse were arrested for fully five minutes; artificial respiration was performed, head and chest lowered, and hypodermics of whiskey administered. Probably ten to fifteen minutes passed until the respiration and pulse became normal and the operation could be continued. On introducing my hand into the pelvis, I found one large mass from which neither uterus, ovaries nor tubes could be distinguished. After a great deal of trouble, I succeeded in freeing the right tube and ovary from their adhesions; both contained pus cavities. The left tube was also removed with the greatest difficulty; its ovary, however, could not be found. A drainage tube was inserted. Twenty-six hours after the operation, a fecal odor was detected in the discharge from the drainage tube. The following day she commenced to discharge fecal matter in large quantities, and from now on most of her feces passed through the tube and on the sides of it, continuing to do so for a week. The wound was kept clean by enemata, which was immediately returned through the fistulous opening. During this time her appetite was poor, vomiting very frequent, so that she became exceedingly weak. The fistula gradually closed up, so that at the time of her discharge from the hospital, there was only (sometimes at intervals of several days) a slight discharge of flatus. The occurrence of this fecal fistula can easily be explained by the fact that the left tube distended with pus had become adherent to the rectum and discharged its contents through the latter. In separating this tube from its old adhesions to the rectum, the old rectal fistula of necessity was reopened, and as a consequence, by virtue of the life-saving drainage tube, the fecal matter found it way through the external wound.

CASE III.—Pyosalpynx. Mrs. A., three years married, aged 30 years, had one child at eight months, and was never in good health since. Had three attacks of pelvic peritonitis since; with the last one she was brought into Mercy Hospital. A very tender mass could be felt on both sides of her uterus, which was diagnosed as a double pyosalpynx. Laparotomy, April 29th. Tubes and ovaries on both sides very firmly bound down to pelvic floor and adherent to loops of intestines. They were brought up with considerable difficulty and tied off. Ovaries on both sides were firmly attached to their corresponding tubes, each ovary and tube forming one abscess-sac. Drainage. Patient rallied badly from the operation, and vomited incessantly. She had taken ether very badly, a very large quantity being needed to keep her relaxed. The incessant vomiting was therefore attributed to the ether. It kept up for forty-eight hours; for thirty hours her pulse was about 160 and very feeble; had Cheyne-Stokes respiration and an ashy color about her face. She was fed and stimulated freely by rectum, and forty-eight hours after operation she was much improved, her convalescence being then uninterrupted by elevation of temperature, and with a good pulse; appetite was good, and she was sitting up in bed, when on the evening of the twelfth day a sudden change came over her; she complained of pain about the chest and of loss of appetite. Had shown symptoms of hysteria a day or two previous to this. I examined her carefully; her pulse was 100; temperature perfectly normal; abdomen flat without the slightest tenderness. She rested well during the early part of the night, but towards morning she became very restless, and died suddenly at 6 A.M. on the thirteenth day after the operation. A *post mortem* examination could not be obtained. Her death was entirely unexpected and its cause very obscure, though I have reasons to suspect pulmonary embolism.

CASE IV.—Solid tumor of right ovary and ascites. Mrs. S., aged 45, no childen, consulted me about an abdominal tumor, situated on right side of uterus, hard, irregular, freely movable and reaching midway between the anterior superior spinous process of the ilium and the umbilicus. Lately she had suffered so much pain, that she had to be kept under the influence of opiates constantly. The diagnosis was either solid tumor of right ovary, or sub-peritoneal fibroid of uterus with long pedicle. Laparotomy, May 6th. On opening abdomen a considerable quantity of ascitic fluid escaped. The operation was extremely simple as there were no adhesions whatsoever. Patient made an uninterrupted recovery, the temperature never being above normal.

The tumor, of the size of a large cocoanut, had become partly cystic and appears to be a fibroid of the right ovary.

CASE V.—Abscess of left ovary. Miss Annie C., aged 24 years, has been in bad health for over a year. I was called to see her about a year ago, and found a large mass to the left of her uterus, not very tender to the touch, and fluctuating. She was greatly reduced in flesh and very weak. Under treatment her general condition improved, but her local trouble remained the same, though local treatment was conscientiously employed for four months. I then advised laparotomy following my diagnosis of left pyosalpynx, but the operation was refused. She then placed herself under the care of another physician. Two or three months later, while going to her physician's office, on getting off the street car an abscess ruptured through her vagina, and this was followed by a severe attack of pelvic peritonits. At this time I was again called in, and found the mass on her left side considerably larger and exceedingly tender. She was now very anxious to have the opperation performed. Laparotomy, June 16th. Removed a large ovarian abscess containing about a pint of puss. The sac was adherent to the omentum, intestines, anterior surface of bladder, and had to be peeled out; on doing this the tube, firmly attached to it and to the pelvic floor was broken off; the remaining portion was then brought up with great difficulty. The ovary was one large abscess-cavity, and the tube also contained pus. The right tube and ovary were, contrary to the usual rule, not removed, as the patient's condition was such as to make it dangerous to perform a second operation on her, especially as they seemed perfectly healthy. In breaking up the adhesions around the abscess-sac I accidently ruptured it, and the pus poured freely into the abdominal cavity. This was thoroughly and repeatedly washed out with hot water and a drainage tube introduced.

Her recovery was uninterrupted; the temperature and pulse remaining perfectly normal, except on the day after the operation, when the temperature rose to 100. She is now in perfect health and has gained much in flesh.

